# Electrical Characteristics of Polypropylene Mixed with Natural Nanoclay

**DOI:** 10.3390/polym10090942

**Published:** 2018-08-24

**Authors:** Huseyin R. Hiziroglu, Iosif E. Shkolnik

**Affiliations:** Department of Electrical & Computer Engineering, Kettering University, Flint, MI 48504, USA; shkolniki@comcast.net

**Keywords:** breakdown, isotactic polypropylene, nanocomposites, natural nanoclay

## Abstract

Polypropylene has been used in radio-frequency capacitors and has also started to be employed in cables as insulation. The objective of this study was to evaluate the electrical properties of polypropylene filled with natural clay as a nano-material. Polypropylene samples having 0%, 2% and 6% natural clay by weight were exposed to 60-Hz sinusoidal voltages at two different rates of rise. The breakdown voltage of each sample was recorded at these different ramp rates. Also, the Root-mean-squared (rms) current was measured as the voltage was increased across the test samples. The important findings of this study were (a) the breakdown strength of the natural nanoclay-filled polypropylene was higher than the unfilled polypropylene, and the optimum concentration of nanoclay appeared to be 2% by weight; (b) the current density as a function of the electric-field intensity indicated a non-linear behavior with saturation, and the saturation onset took place at a higher electric-field intensity in nanoclay-filled polypropylene, wherein 2% nanoclay seemed to be the optimum concentration as well for the onset electric field of saturation.

## 1. Introduction

Nano-particles have found a wide variety of applications in order to modify the properties of polymer dielectrics. Since the beginning of this millennium, studies into nanocomposites with polymer-based materials for electrical applications as dielectrics and insulators have been intensified considerably in order to better understand various phenomena in these materials so that their effective usage will be possible in numerous applications [1].

It is known that the addition of nano-particles to a polymer seems to enhance the overall properties of the composite [1]. In such a nanocomposite, interfaces between two phases, one being a nanoparticle and the other being the host polymer, play a significant role [2] in its properties. This is primarily due to the fact that the volume of the interfaces dominates the whole volume of the composite as the size of the nanoparticle decreases [2]. This effect could also substantially modify the overall dielectric characteristics of the composite material [2]. Thus, studies into polymer-based nanocomposites have been underway in order to use them in high-voltage cables as an insulating material [3]. At present, polymeric materials used in cables in high-voltage applications are primarily various versions of polyethylene (PE), with cross-linked polyethylene (XLPE) being one of the most widely used [4]. With the present technologies the most powerful extruded high-voltage cables with PE as an insulating material used in high-voltage, direct-current (HVDC) systems can handle up to 640 kV [3].

A polymeric nanocomposite based on PE filled with nanometric size metal-oxide has been introduced recently due to the lower charge mobility than that of pure PE as a potential candidate for HVDC cable insulation [3]. Also, a combination of PE and metal-oxide nano-particles seemed to be promising in ultra-high-voltage applications due to the interface role of the nanomaterial in the host material [3]. Therefore, it is a fact that polymer-based nano-dielectrics have shown not only enhanced electrical properties but also indicated better mechanical and thermal properties than the plain polymeric material. Also, various studies have revealed that nanocomposites with polymer dielectrics have exhibited significant improvements in optical and physico-chemical properties [5].

Along with PE and other polymers, polypropylene (PP) has found a place in electrical systems as a dielectric [6]. The isotactic form of PP has a melting temperature of 481 K with high crystallinity that leads to having high stiffness and tensile strength [7]. Although isotactic PP has high crystallinity it is considered to be a semicrystalline material since crystallization of PP is a random process influenced by the probability of a molecule’s ability to develop a crystalline structure with adjacent molecules [7]. Therefore, crystalline phase, amorphous phase and crystalline/amorphous interface have importance in the properties of charge transfer in PP similar to the phenomena taking place in PE as described in [3,8]. Due to these highly desirable properties, PP films are used as the dielectric medium in high-performance pulse and low-loss RF capacitors [9,10]. Also, PP has been employed as an insulating medium in power cables [11] primarily due to its relatively high melting temperature that might possibly facilitate higher current-carrying capacity [12] in addition to other such microstructure properties as crystallinity, amorphous phase as well as crystalline/amorphous interface [3]. Moreover, it should be noted that an appropriate process such as extrusion of cables should be selected in order to optimize the mechanical and electrical strengths since they could be influenced by the crystallinity and crystal thickness in the material [3,13,14].

Different electrical properties of PP have been investigated in various studies [9,10,11,15,16,17]. One of the studies done with PP was to evaluate its dielectric spectrum at low voltages [18,19]. Another work was the experimental investigation of the space charge distribution in PP and its nanocomposites [20]. In fact, improvements have been observed in the space-charge distribution when PP was loaded with natural clay. Consequently, it is hypothesized that the breakdown behavior of PP could also improve when filled with nano-material. However, there is very little information on the electrical breakdown [21] and the current density in PP mixtures with nano-particles and it needs to be further studied. Therefore, the objectives of this study are to evaluate and compare; (a) the breakdown strength and (b) the variation of current-density as a function of applied electric-field intensity of PP-based nanocomposite films with various contents of nano-size natural clay in PP. It is expected that the initial data from this work could be beneficial to better understand PP loaded with 2 and 6 wt % natural clay so that they can be used with enhanced properties in different applications in the electrical industry.

## 2. Materials and Methods

### 2.1. Materials

The polyolefin used in this study is isotactic PP, and the natural nanoclay is montmorillonite, [Al_l.67_ Mg_0.33_ (Na_0.33_)]Si_4_ O_l0_ (OH)_2_. Since PP is a hydrophobic material and purified natural clay is hydrophilic, they are immiscible thermodynamically [22]. Consequently, natural nanoclay needs to be intercalated and later in the process compatibilized [22]. After the intercalation process of montmorillonite with dimethyl di(hydrogenated tallow) ammonium chloride, also known by its trade name as Cloisite^®^20A [22,23], the natural nanoclay turned into hydrophobic from hydrophilic. In the next step, compatibilization was done using Polybond^®^3150 with a composition of PP-maleic anhydride modifier (PP-MA) [22]. Also included in the batch was Irganox^®^B 225, an antioxidant [22], with a blend of 50% tris(2,4-ditert-butylphenyl)phosphite and 50% pentaerythritol tetrakis[3-[3,5-di-tert-butyl-4-hydroxyphenyl]propionate], in order to avoid the degradation of the nanocomposite due to oxidation, which is very important especially when used as cable insulation [3]. After being pelletized, the nanocomposite was brought to 135 μm by a film blowing technique at 180 °C. Then, the films were rolled at 115 °C. Further details of this material preparation were presented in a paper from National Research Council of Canada [22], the institution that prepared and supplied the samples for this study. In this experimental study, three sets of samples were used. Sample 1 was isotactic PP with the trade name of Pro-fax HL-451H from Basell as the control sample. Its composition was 87 wt % PP, 0.2 wt % antioxidants and 12.8 wt % compatibilizer. Sample 2 was a nanocomposite with a composition of 85.3 wt % PP, 0.2 wt % antioxidants, 12 wt % compatibilizer and 2 wt % nanoclay. Finally, Sample 3 was also a nanocomposite having 81.8 wt % PP, 0.2 wt % antioxidants, 12 wt % compatibilizer and 6 wt % nanoclay.

Each nanocomposite sample had a thickness of 135 μm with a variation of ±10%. Before being situated between two stainless-steel electrodes, the thickness of each sample was recorded using a micrometer. Each electrode had a circular flat surface with a diameter of 33 mm and rounded edges in order to minimize electric-field non-uniformity. The electrode-film assembly was immersed into highly refined transformer oil in a stainless-steel test cell.

### 2.2. Methods

**Figure 1:**
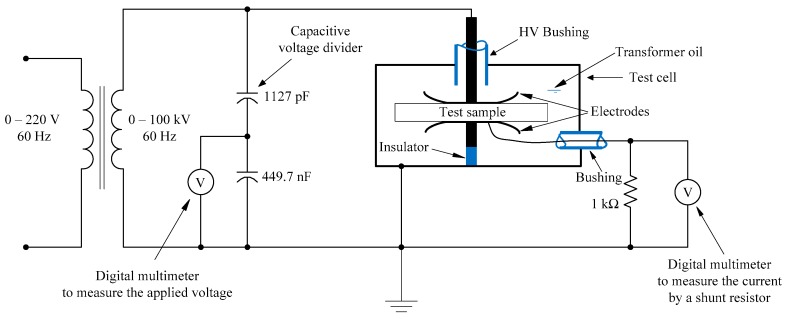
Principle circuit schematic of the experimental setup.

Figure 1 shows the experimental test apparatus that consisted of a stainless-steel test cell and a high-voltage transformer with a model number of TEO 100/10 from Messwandler Bau, Germany, along with auxiliary elements in order to make the measurements possible.

Because the thickness of the test sample is much smaller than the diameter of the electrodes, the electric-field distribution in the test sample can be considered as almost uniform. Also, in order to avoid the possibility of breakdown in air around the electrode edges, the assembly of the electrodes and the test sample were immersed in highly refined transformer oil filling the test cell.

The rating of the high-voltage test transformer was 220/100,000 V (rms) with output power of 5 kVA at 60-Hz.

A capacitive voltage divider CM from Messwandler Bau, Germany, was incorporated in order to measure the applied rms voltage to the test sample as indicated in Figure 1. The accuracy of the voltmeter (34405A, Agilent Technologies, Santa Clara, CA, USA) connected to the low-voltage arm of the voltage divider was better than 1%. A 1-kΩ precision resistor with a tolerance of ± 0.01% was connected between the low-voltage electrode and the ground as shown in Figure 1 since the rms current in the circuit is directly proportional to the rms voltage across 1-kΩ resistor. A voltmeter (34401A, Agilent Technologies, Santa Clara, CA, USA) having an acV accuracy of less than 0.1% was employed to measure the rms voltage across the 1-kΩ resistor. The applied voltage across the test sample was a 60-Hz sinusoidal waveform and was raised from zero volt up to the voltage at which the failure occurred in the sample. While increasing the voltage at a rate of rise of 90 V/s, typically 15 to 20 data points were recorded for the voltage and current from zero up to the breakdown voltage. From the experimental results, electric-field intensity and the current density were calculated. In order to verify the reproducibility of the experimental results experiments were performed on 5 different samples with the same amount of nanofiller. Moreover, the breakdown voltages were also recorded with a voltage rate of rise of 1050 V/s for assessing if there were significant changes in the breakdown voltages. These breakdown tests were also performed on 5 different samples with the same nanofiller content.

## 3. Results

### 3.1. Breakdown Strength

**Figure 2:**
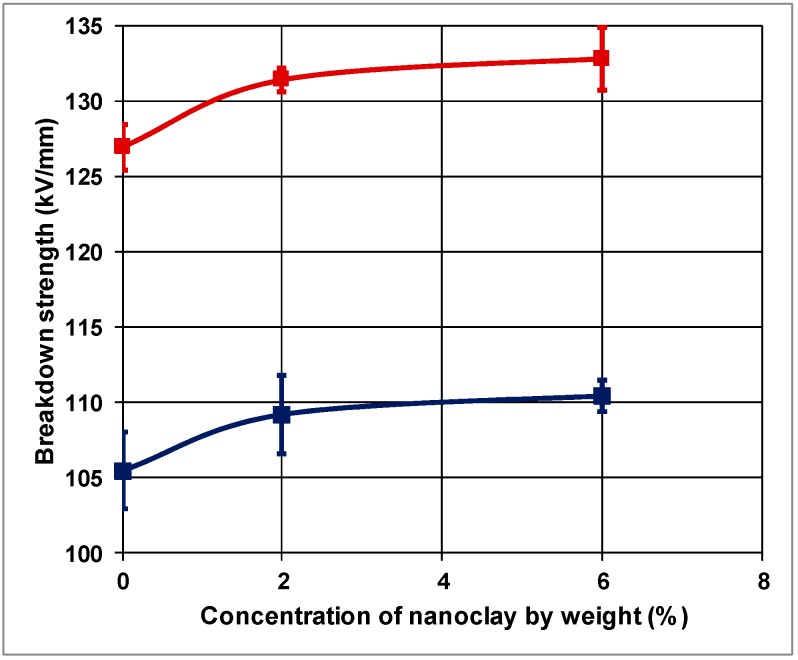
Breakdown strength vs. the concentration of natural clay in PP and its nanocomposites with voltage rate of rise being parameter [21]. 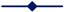 90 V/s; 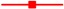 1050 V/s.

Since the electric field has almost a uniform distribution in the samples, the breakdown strength can be calculated as:

*E*_b_ = *V*_b_/*d* (1)

where *E*_b_ is the breakdown strength, *V*_b_ is the breakdown voltage and *d* is the thickness of the sample. Figure 2 exhibits the trend of the mean breakdown strengths of the PP and its mixtures with different concentrations of natural nanoclay.

As can be seen from Figure 2, the mean breakdown strength of five PP samples was evaluated as 105.5 kV/mm with a standard deviation of 5.7 kV/mm at 90 V/s voltage rate of rise.

In Figure 2, the bars on the data points indicate the standard error of the mean that was determined as 2.55 kV/mm. The mean breakdown strength of nanocomposite with a concentration of 2 wt % natural clay, however, was 109.2 kV/mm with a standard deviation of 5.8 kV/mm and a standard error of 2.6 kV/mm as shown in Figure 2. There is an improvement of about 3.5% on the breakdown strength of nanocomposite with 2 wt % of natural clay concentration with respect to the control PP. The mean breakdown strength of PP loaded with 6 wt % natural clay was 110.5 kV/mm with a standard deviation of 2.4 kV/mm and a standard error of 1.06 kV/mm. The improvement in the breakdown strength was 4.7% with respect to the control PP, while the improvement was only about 1% with respect to the nanocomposite with 2% concentration of natural clay.

Experimental procedures were the same when a ramp speed of 1050 V/s was used as with the 90 V/s rate of rise in order to assess the breakdown strength of the test samples. At this ramp speed, the breakdown strength of 5 samples is also presented in Figure 2 with different nanoclay contents in PP.

Figure 2 reveals that the control PP has the smallest breakdown strength as compared to the PP-based composites. The mean value of the breakdown strength of the control PP is 127 kV/mm. The standard deviation and the standard error are, as indicated in Figure 2 with error bars, found to be 3.5 and 1.5 kV/mm, respectively. The mean breakdown strength of PP-based nanocomposite loaded with 2 wt % natural nanoclay is determined as 132 kV/mm. For this mean value 1.7 and 0.8 kV/mm are, respectively, the standard deviation and the standard error. The PP nanocomposite with 6 wt % nanoclay content, however, has a mean breakdown strength of 133 kV/mm. The standard deviation and standard error, for this case, are determined as 4.7 and 2.1 kV/mm, respectively.

At a higher ramp-speed of 1050 V/s, nanocomposite with 2 wt % nanoclay content indicated about 3.9% improvement on the breakdown strength as compared to control PP that has no natural clay content. The increase in breakdown strength, however, was not, once again, as substantial when the nanoclay content was increased from 2 to 6 wt %. When compared, the variations of the breakdown strengths as a function of the natural clay concentration in PP at two different rates of rise of the applied voltage, in Figure 2, it is apparent that the breakdown strength increases when the ramp speed is increased from 90 to 1050 V/s. The data presented in Figure 2 have a confidence level of 95% on the error bars.

### 3.2. Characteristics on the Variation of Current Density as a Function of Electric-Field Intensity

The current-density vs. electric-field intensity characteristics of PP and its nanocomposites with natural clay were also evaluated within the scope of this study. In this context, current density essentially corresponds to the applied current injected to the sample. Due to the nature of dielectrics, applied current can be modelled with two components under sinusoidal steady-state. These currents are the conduction current due to the transport of charge, and the displacement current due to the rate-of-the-change of electric-flux density with respect to time in the material. Using a Tektronix oscilloscope (TBS 1052B, Tektronix, Beaverton, OR, USA) the current leads the applied voltage by 1.5359 rad at 60-Hz. Because this angle is close to π/2 rad, which is for perfect dielectric, it is apparent that the conduction current is insignificant as compared to the displacement current. Thus, the conduction current at 60-Hz is neglected and the measured current is considered to be approximately the displacement current. In fact, the amount of error in the current introduced with this approximation was calculated ±0.061%. Therefore, the current density is primarily governed by the permittivity of the material under consideration.

*J* = *I*/*A* (2)

**Figure 3:**
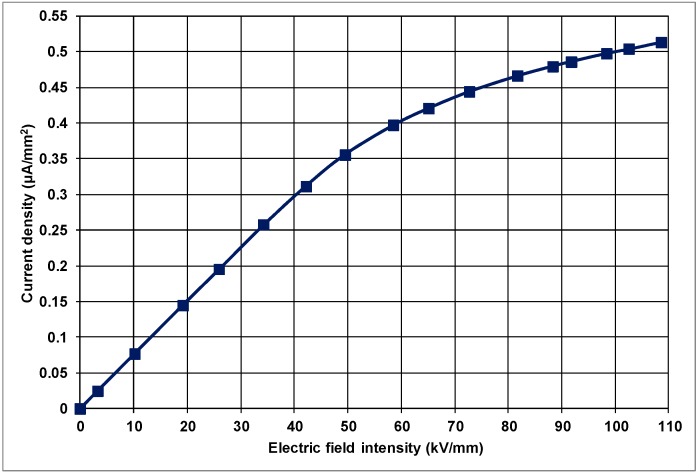
Variation of current density with electric-field intensity in unfilled PP.

Here, *J* is the current density (A/m^2^), *I* is the applied current to the material and *A* is the surface area of the material effectively sandwiched between the electrodes (m^2^). Figure 3 presents the current-density vs. applied electric-field intensity for the plain PP as a control sample. The graph in Figure 3 points out that there is a steep linear increase in current density at lower electric fields. Although the current density continues to increase above the applied electric-field intensity of about 30 kV/mm, this increase is no longer as steep as it was below 30 kV/mm. Therefore, the *J* vs. *E* characteristic is non-linear, with saturation for the control PP.

From Maxwell’s equations, the displacement current density is directly proportional to the electric-flux density in frequency domain as:

*J* = *ωD* (3)

where *ω* is the angular frequency (rad/s) and *D* is the electric flux density vector (C/m^2^).

Also, due to the constitutive properties of dielectric materials, electric flux density is directly related to the permittivity of the medium along with the applied electric-field intensity as:

*D* = *εE* (4)

Here, *E* is the applied electric-field intensity (V/m) and *ε* is the permittivity of the medium (F/m), which can also be expressed as:

(5) $$\varepsilon =\varepsilon_{o}(1+\chi_{e})$$

with *ε*_o_ = 8.85 × 10^−12^ F/m being the free-space permittivity and $\chi_{e}$ being the electric susceptibility that is proportional to the polarization vector,

(6) $$P=\varepsilon_{o}\chi_{e}E$$

of the material [24]. If the material is polarizable with electric field, the permittivity will be larger than the free-space permittivity.

Relative permittivity, which is often referred to as the dielectric constant, is also defined as:

*ε*_r_ = *ε*/*ε*_o_ (7)

**Figure 4:**
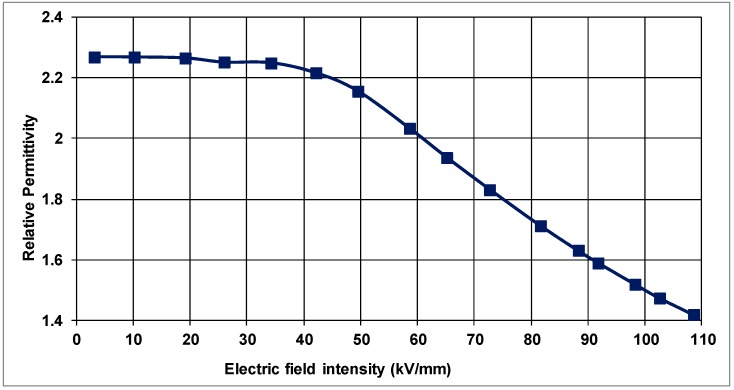
Relative permittivity as a function of electric-field in unfilled PP.

For the unloaded PP, raising the electric-field intensity to 30 kV/mm from zero yields a permittivity of 2.27, which is nearly constant, as shown in Figure 4. From the same figure, at higher electric fields than 30 kV/mm, however, the relative permittivity diminishes to about 1.46 at 110 kV/mm.

It should also be noted that these values may differ from one kind of PP to another since the composition may have different concentrations of PP along with different component materials. Nevertheless, the relative permittivity of PP under consideration is within the limits of the dielectric constant (2.2–2.5) in the linear region of the *J* vs. *E* characteristic as stated in standards [25].

**Figure 5:**
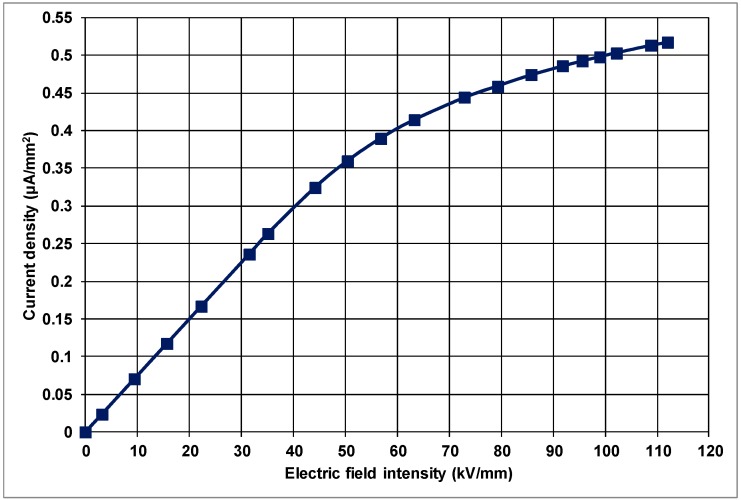
Variation of current-density with electric-field intensity in PP composite filled with 2 wt % natural nanoclay.

The variation of current density with the electric-field intensity is given in Figure 5 for PP doped with 2 wt % nanoclay.

**Figure 6:**
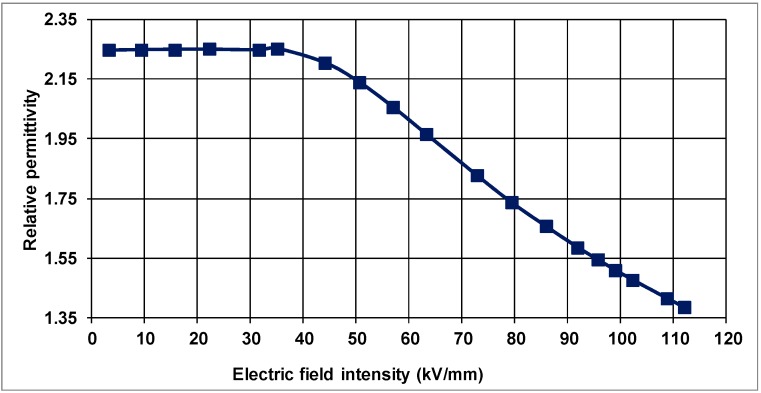
Relative permittivity as a function of electric-field in PP composite filled with 2 wt % natural nanoclay.

It can be observed from Figure 5 that the current density varies with applied electric-field intensity in the nanocomposite similar to that of the unfilled, control PP samples, and is non-linear for the electric-field intensities of 35 kV/mm and above. The relative permittivity, on the other hand, remains constant around 2.25 up to about 35 kV/mm in PP filled with 2 wt % natural clay as shown in Figure 6 for the linear region of the *J*–*E* characteristic of the material. For electric fields higher than 35 kV/mm, however, there is a substantial reduction on the relative permittivity from 2.25 to 1.38 at 110 kV/mm due to the non-linearity of the material.

**Figure 7:**
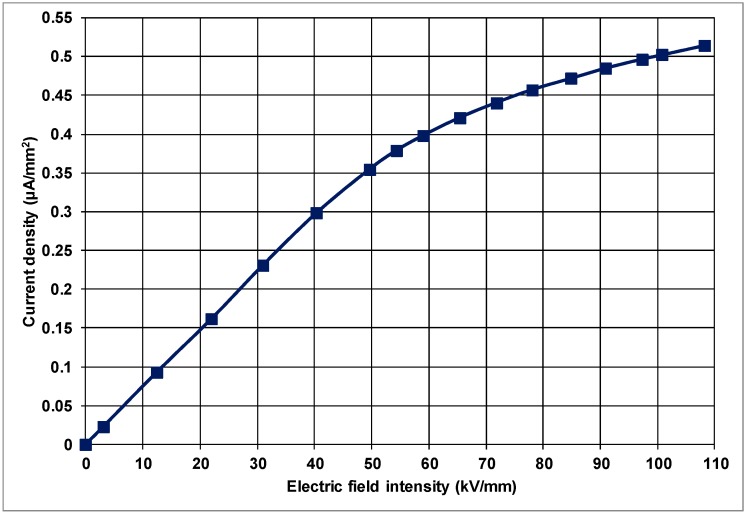
Variation of current-density with electric-field intensity in PP composite filled with 6 wt % natural nanoclay.

PP filled with 6 wt % natural clay also revealed a non-linear, saturation-type *J* vs. *E* characteristic as pointed out in Figure 7. The displacement-current density started to saturate at approximately 33 kV/mm.

**Figure 8:**
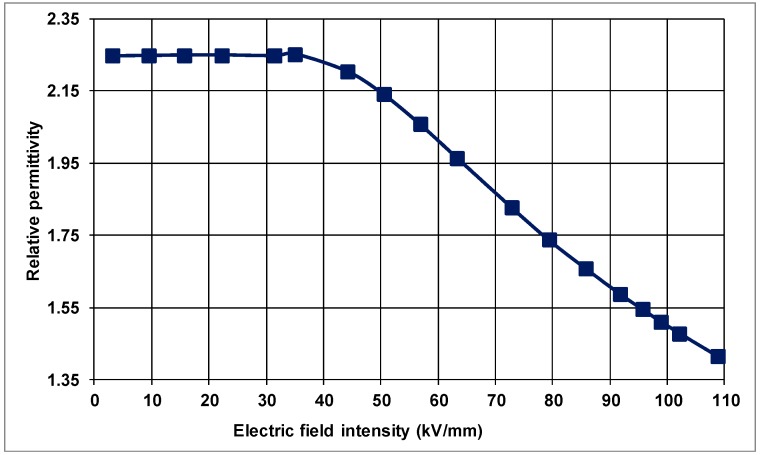
Relative permittivity as a function of the electric-field in PP composite filled with 6 wt % natural nanoclay.

The relative permittivity of this nanocomposite appears to be nearly constant at 2.24 up to the electric-field intensity of 33 kV/mm as presented in Figure 8.

Similar to the plain PP and the nanocomposite filled with 2 wt % nanoclay, the material exhibits a reduction in its relative permittivity to 1.42 at 110 kV/mm. As can be observed from these results, natural nanoclay in PP did not contribute significantly to the current density vs. electric-field characteristic or to the relative permittivity.

## 4. Discussion

The dielectric breakdown of PP is affected by the polymer morphology when PP is subjected to sinusoidal voltage. PP usually breaks down electrically in locations where the material is less dense. Andritsch et al. indicated that the smallest breakdown voltages took place in the interspherulitic zones of PP, whereas the highest breakdown voltages took place in the spherulites regions [12]. Therefore, the improvement observed in the breakdown strength of nanocomposites of PP could be due to the natural nanoclay that fills the regions less dense in PP.

Also, an optimum content of nanoclay is proposed according to the results on the breakdown strengths in this investigation. It was observed that the breakdown strength did not improve substantially for the nanocomposite filled with 6 wt % nanoclay when compared to 2 wt %. Therefore, nanocomposite loaded with 2 wt % natural clay was considered to be the optimum amount of natural clay. Abou-Dakka et al. concluded similarly with the similar nanocomposites of PP in which all the homocharges could possibly be reduced for the optimum nanoclay content [11].

When the rate of rise of the applied voltage was increased from 90 to 1050 V/s across unfilled PP and its nanocomposite samples, a significant increase was observed on the breakdown strength of the materials. The increase in breakdown strength is due to the insufficient time to transfer energy to the particles in the unfilled PP as well as in the natural nanoclay-filled samples to initiate the ionization activity when the rate of rise of the applied voltage is higher. Thus, a higher electric-field intensity is required to maintain higher energy for the inception of ionization activity that might possibly cause a sustained discharge leading to the failure of the material.

The saturation effect in the current density vs. electric-field intensity characteristics in unfilled PP and its nanocomposites loaded with natural nanoclay can be explained based on the net polarization of the materials. From Equation (3) it should be noted that the electric flux density is an angular frequency reduced displacement current under sinusoidal steady state. Also, the electric flux density is proportional to the net polarization of the material as indicated in Equations (4)–(6). When the applied electric field is raised starting from zero up to a certain level, the displacement current density increases linearly since it is directly proportional to the net polarization in the material. Above that certain electric-field intensity, on the other hand, the rate-of-change of net polarization with respect to the electric-field intensity becomes lower. Consequently, the saturation effect starts occurring in the material. In the linear region of the *J*–*E* characteristics of the materials, the relative permittivity remains almost constant since the net polarization of the material takes place at a constant rate of change with respect to the electric field. But, with respect to a higher electric field, net enhancement rate of polarization diminishes and leads to a significant reduction in the permittivity of the material. In a another work it was also stated that although polymeric materials have almost constant permittivity at relatively low electric fields, at high electric fields, substantial drops could be seen in the permittivity [26].

## 5. Conclusions and Future Work

As discussed in Section 4, one of the important conclusions of this study was, PP loaded with 2 and 6 wt % natural nanoclay indicated an improved breakdown strength when compared to the unfilled isotactic PP. However, it was observed that breakdown strength improved significantly as soon as the PP was doped with 2 wt % natural nanoclay. When the nanoclay content increased to 6 wt % there was still an increase in breakdown strength, but not as sharp as it was from plain PP to 2 wt % nanoclay loaded PP. Also, as the ramp speed of the applied voltage increased, breakdown strength of all the materials under consideration increased.

Current density vs. electric-field intensity characteristics of all the materials in this study have shown very similar trends with some saturation effect. When the electric field was raised in the material up to a certain level, it seemed to have a linear behavior. However, above a critical applied electric field, materials responded non-linearly as discussed in Section 4. This was essentially due to the reduction of the enhancement-rate of the net electric polarization in the PP as well as its composites with nanoclay.

Surface modification seems to be important for the contribution of the filler material to the properties of nanocomposites as indicated in [3,13,14,27]. As a future study, the influence of surface modification could be investigated on the breakdown and permittivity properties of the PP loaded with natural nanoclay. Also, further study should be performed on the aging of nanocomposites of PP with natural clay since their life prediction is important especially when used in capacitors and cables.
